# Networking the host immune response in *Plasmodium vivax* malaria

**DOI:** 10.1186/1475-2875-12-69

**Published:** 2013-02-21

**Authors:** Vitor RR Mendonça, Artur TL Queiroz, Fabrício M Lopes, Bruno B Andrade, Manoel Barral-Netto

**Affiliations:** 1 Centro de Pesquisas Gonçalo Moniz, Fundação Oswaldo Cruz, Salvador, Brazil; 2 Faculdade de Medicina, Universidade Federal da Bahia, Salvador, Brazil; 3 Instituto de Matemática e Estatística, Universidade de São Paulo, São Paulo, Brazil; 4 Coordenação de Informática, Universidade Tecnológica Federal do Paraná, Cornélio Procópio, Brazil; 5 Immunobiology Section, Laboratory of Parasitic Diseases, National Institute of Allergy and Infectious Diseases, National Institutes of Health, 20893, Bethesda, MD, USA; 6 Instituto de Investigação em Imunologia, Instituto Nacional de Ciência e Tecnologia, São Paulo, Brazil

**Keywords:** Malaria, *Plasmodium vivax*, Biomarkers, Network analysis

## Abstract

**Background:**

*Plasmodium vivax* malaria clinical outcomes are a consequence of the interaction of multiple parasite, environmental and host factors. The host molecular and genetic determinants driving susceptibility to disease severity in this infection are largely unknown. Here, a network analysis of large-scale data from a significant number of individuals with different clinical presentations of *P. vivax* malaria was performed in an attempt to identify patterns of association between various candidate biomarkers and the clinical outcomes.

**Methods:**

A retrospective analysis of 530 individuals from the Brazilian Amazon, including *P. vivax*-infected individuals who developed different clinical outcomes (148 asymptomatic malaria, 187 symptomatic malaria, 13 severe non-lethal malaria, and six severe lethal malaria) as well as 176 non-infected controls, was performed. Plasma levels of liver transaminases, bilirubins, creatinine, fibrinogen, C-reactive protein, superoxide dismutase (SOD)-1, haem oxygenase (HO)-1 and a panel composed by multiple cytokines and chemokines were measured and compared between the different clinical groups using network analysis.

**Results:**

Non-infected individuals displayed several statistically significant interactions in the networks, including associations between the levels of IL-10 and IL-4 with the chemokine CXCL9. Individuals with asymptomatic malaria displayed multiple significant interactions involving IL-4. Subjects with mild or severe non-lethal malaria displayed substantial loss of interactions in the networks and TNF had significant associations more frequently with other parameters. Cases of lethal *P. vivax* malaria infection were associated with significant interactions between TNF ALT, HO-1 and SOD-1.

**Conclusions:**

The findings imply that clinical immunity to *P. vivax* malaria is associated with multiple significant interactions in the network, mostly involving IL-4, while lethality is linked to a systematic reduction of complexity of these interactions and to an increase in connections between markers linked to haemolysis-induced damage.

## Background

The vast majority of human diseases do not result from single molecular changes, and malaria is certainly included in this group. Besides factors related to the *Plasmodium* parasite itself, malaria is a consequence of multiple host molecular and genetic alterations compounded by environmental factors [1]. Most of the studies in malaria immunopathogenesis are focused on the identification of one or a small group of candidate molecules related to disease severity. The cytokine balance seems to influence malaria outcome, with TNF, IFN-γ and IL-10 emerging as key players in both experimental models and in human observational studies [2-6]. Recently, host factors associated with oxidative stress, such as the enzymes superoxide dismutase 1 (SOD-1) and haem oxygenase (HO)-1, as well as molecules that are linked to metabolic adaptation to iron overload during malaria-triggered haemolysis, have been identified as very potent biomarkers to distinguish individuals developing more severe forms of vivax malaria [7-9]. However, the patterns of interaction between these multiple factors within the clinical spectrum of human malaria are largely unexplored. The situation is even worse concerning the infection caused by *Plasmodium vivax*, which has been relatively neglected for many years and recently re-emerged as a potentially lethal disease [6,10-13].

The use of a comprehensive approach to exploring the relationship between groups of molecules likely to be related to the severity of the disease manifestations may contribute to a better understanding of the patterns of susceptibility to disease severity or disease tolerance. In this study, network analysis of a large dataset from a significant number of individuals presenting with different clinical manifestations of vivax malaria was performed in an attempt to identify patterns of association between various candidate biomarkers and the clinical outcomes. The findings from this exploratory study demonstrate a new level of complexity connecting several mediators involved in the immunopathogenesis of vivax malaria.

## Methods

### Study design and participants

The present study is a retrospective analysis of a databank containing clinical, epidemiological and immunological data from 530 individuals from the Brazilian Amazon (Rondônia, Brazil) recruited between 2006 and 2007, as part of a project aimed at describing determinants of susceptibility to vivax malaria that was finalized on 2010. In this project, both active and passive malaria case detection were performed. These included home visits in areas of high transmission (active case detection), and study of individuals seeking care at the diagnostic centres of Brazilian National Foundation of Health (FUNASA) or in a municipal hospital in Buritis, Rondônia, Brazil (passive case detection). Individuals of both sexes, ranging in age from five to 70 years, who had resided in the endemic area for more than six months, were invited to participate. The details of the recruitment, diagnosis approach and clinical definitions of asymptomatic infection or severe malaria caused by *P. vivax* used in the project were published previously [6-9,14-17]. The exclusion criteria were as follows: *Plasmodium falciparum* infection documented by both microscopy and nested polymerase chain reaction (PCR), documented viral hepatitis, chronic alcoholism, human immunodeficiency virus type 1 infection, yellow fever, leptospirosis, cancer and chronic degenerative diseases, sickle cell trait and the use of hepatotoxic or immunosuppressant drugs. The malaria diagnosis was made in a reference centre from the National Foundation of Health in the endemic area and confirmed with nested PCR as previously described [14,15,17]. Individuals without symptoms were actively recruited in their residencies by active search, mostly in remote riverine communities and had thick blood smear samples and a small aliquot of blood in EDTA tubes collected for diagnostic screening by nested PCR. The diagnosis in those individuals was performed after the clinical visit and if a positive test for malaria was found, a second visit within 30 days was performed in order to search for appearance of malaria symptoms. A new sample collection was made and a second round of diagnostic tests was performed. Subjects that remained with positive nested PCR for *Plasmodium* within this period of 30 days and presented no malaria-related symptoms, such as fever (axillary temperature >37.8°C), chills, sweats, myalgia, arthralgia, strong headaches, nausea, vomiting, jaundice, and severe asthenia during this period were considered as asymptomatic malaria cases. During the first clinical visit (previously to the results of the diagnostic tests) all the asymptomatic individuals received counselling and were oriented to seek for health care in a malaria reference centre in case symptoms appeared during the period between the 2 clinical visits. After the second clinical visit, all the patients that had positive malaria screening with microscopy and/or nested PCR were treated following the treatment guidelines of the Brazilian Ministry of Health. The plasma samples used to assess the biomarkers were collected during the second clinical visit, before the initiation of anti-malarial drugs. Symptomatic individuals were promptly treated. Data from a total of 530 individuals were analysed, and the individuals were stratified into the following categories: non-infected (n=176), asymptomatic vivax malaria (n=148), symptomatic vivax malaria (n=187), severe non-lethal vivax malaria (n=13), and severe vivax malaria associated with mortality (n=6). The plasma samples obtained from patients with severe forms of malaria, including the cases that eventually died during the follow up were collected at the hospital admission and the procedures are described elsewhere [6]. Demographic characteristics of the study participants are shown in the Table 1.

**Table 1:** Demographic characteristics of the participants

	**Endemic controls**	**Asymptomatic malaria**	**Symptomatic malaria**	**Severe malaria**		**P-value**
				**Survivors**	**Deaths**	
	**n=176**	**n=148**	**n=187**	**n=13**	**n=6**	
Male (%)	72 (40.9)	70 (47.3)	93 (49.7)	7 (53.8)	3 (50.0)	0.504
Age - years						<0.001
Median	32	40	33	22	27	
IQR	24-45	32-49	27-42	16-30	13-44	
Years residing in the area						<0.001
Median	12.6	11.8	7.6	2.6	3	
IQR	3.2-14.8	3.5-16.4	0.6-10.1	0.5-4.8	0.3-5.2	
Parasites/μL						<0.001
<500	176	145	49	0	0	
	(100%)	(98.0%)	(26.2%)			
500-5,000	0	3(2.0%)	84	4	1	
			(44.9%)	(30.8%)	(16.7%)	
5,001-50,000	0	0	50	6	3	
			(26.7%)	(46.1%)	(50%)	
>50,000	0	0	4	3	2	
			(2.1%)	(23.1%)	(33.3%)	

IQR: interquartile range.

### Ethics statement

Written informed consent was obtained from all participants or their legally responsible guardians, and all clinical investigations were conducted according to the principles expressed in the Declaration of Helsinki. The project was approved by the institutional review board of the Faculdade de Medicina, Faculdade São Lucas, Rondônia, Brazil, where the study was performed.

### Laboratory measurements

Several mediators were selected based on the assessment of the overall inflammatory status and immune responses in malaria. All the biomarkers that were measured in all the patients, and were contained in the databank, were included in the analysis and no pre-selection was done. Plasma measurements of aspartate amino-transferase (AST), alanine amino-transaminase (ALT), total bilirubin, direct bilirubin, creatinine, fibrinogen and C-reactive protein (CRP) were made at the clinical laboratory of Faculdade São Lucas and at the Pharmacy School (Federal University of Bahia, Brazil). The cytokines IL-1β, IL-4, IL-6, IL-8, IL-10, IL-12p70, IFN-γ, TNF and the chemokines CCL2 (MCP-1), CCL5 (RANTES), CXCL9 (MIG) and CXCL10 (IP-10) were measured using the cytometric bead array (CBA) (BD Biosciences Pharmingen, San Diego, CA, USA). The flow cytometric assay was performed and analysed by a single operator, and standard curves were derived from cytokine standards. The experiments were performed according to the manufacturers’ instructions. ELISA kits were used to measure the soluble TNF receptor I (sTNF-RI; R&D Systems, Minneapolis, MN, USA), transforming growth factor (TGF)-β (R&R Systems), SOD-1 (Calbiochem, San Diego, CA, USA) and HO-1 (Assay Designs, Ann Arbor, MI, USA) according to the manufacturers’ protocols. In order to compare the distribution of the biomarkers according to the clinical groups, the laboratory parameters were categorized in cytokines (Table 2), surrogates of inflammatory damage (Table 3) and chemokines and other proteins (Table 4).

**Table 2:** **Distribution of cytokines levels in the study subjects stratified by *Plasmodium vivax *malaria clinical outcome**

**Biomarker**	**Endemic controls**	**Asymptomatic malaria**	**Symptomatic malaria**	**Severe malaria: Survivors**	**Severe malaria: Deaths**	**P value**	**P value**
						**1**	**2**
	**n=176**	**n=148**	**n=187**	**n=13**	**n=6**		
IL-1β	5.7	4.0	11.4	7.6	15.54	<0.001	<0.001
pg/mL	(3.5-17)	(2.7-7.8)	(6.2-25.5)	(6.6-23.8)	(4.8-29.3)		
IL-4	23.4	22.1	29.89	30.1	36.4	<0.001	0.008
pg/mL	(12.3-40)	(12-34.4)	(16.8-102)	(18.5-41)	(18-115)		
IL-6	8.4	10.3	69.2	78.5	101.2	<0.001	<0.001
pg/mL	(5.2-20)	(1.5-21.4)	(23.9-105.5)	(56-105)	(41-140)		
IL-8	6.3	3.6	26.0	12.4	66.8	<0.001	<0.001
pg/mL	(4.7-11)	(2.3-9.2)	(5.9-102.5)	(6.1-66.7)	(15-211)		
IL-10	12.0	62.0	125.0	140.2	110.5	<0.001	0.349
pg/mL	(7-20.4)	(11.3-89.6)	(65.3-455.1)	(85-550)	(79-134)		
IL-12p70	7.7	13.5	20.7	12.7	10.0	<0.001	0.520
pg/mL	(4.9-16)	(7.7-18.3)	(12.4-30.5)	(5.1-20.9)	(5-15)		
IFN-γ	32.1	54.3	103.4	212.5	181.6	<0.001	<0.001
pg/mL	(11-62)	(23.6-142.0)	(42.0-324.0)	(80-465)	(65-357)		
TNF-α	0	2.4	38.5	57.2	31.95	<0.001	<0.001
pg/mL	(0–10.5)	(0–10.3)	(18.1-80.5)	(32.5-84)	(19-76.8)		
TGF-β	23.8	89.8	111.1	48.6	40.2	<0.001	<0.001
pg/mL	(1.3-31)	(39.4-193.8)	(84.5-364.7)	(32-58.6)	(26-44.5)		

Note: Data represent median values and interquartile ranges. P value 1 was obtained using Kruskal-Wallis test, whereas P value 2 was calculated using Linear trend post test.

IQR: interquartile range.

**Table 3:** **Assessment of inflammatory damage in the study subjects stratified by *Plasmodium vivax *malaria clinical outcome**

**Biomarker**	**Endemic controls**	**Asymptomatic malaria**	**Symptomatic malaria**	**Severe malaria: Survivors**	**Severe malaria: Deaths**	**P value**	**P value**
						**1**	**2**
	**n=176**	**n=148**	**n=187**	**n=13**	**n=6**		
Total bilirubin mg/dL	0.7	0.8	1.2	1.8	2.1	<0.001	<0.001
	(0.5-1.1)	(0.5-1.2)	(0.8-1.9)	(1.5-2.5)	(1.2-3.2)		
Direct bilirubin mg/dL	0.3	0.4	0.4	0.6	1.1	<0.001	<0.001
	(0.2-0.4)	(0.3-0.8)	(0.3-0.8)	(0.4-1.3)	(0.3-1.7)		
Indirect bilirubin mg/dL	0.4	0.3	0.7	1.1	1.2	<0.001	<0.001
	(0.3-0.6)	(0.2-0.4)	(0.4-1.2)	(0.8-1.3)	(0.9-1.3)		
AST U/L	43.3	56.4	167	201.0	268.4	<0.001	<0.001
	(34-56.3)	(35.5-87.5)	(81.5-506)	(87-302)	(160-340)		
ALT U/L	40.9	44.9	180	201.1	278.8	<0.001	<0.001
	(33.5-54)	(32-69.4)	(123–438)	(190-304)	(175-342.9)		
Creatinine mg/dL	1.2	1.2	1.3	1.7	2.4	<0.001	<0.001
	(1.1-1.3)	(1.0-1.3)	(1.2-1.4)	(1.3-2.5)	(1.9-2.5)		
CRP mg/L	5.2	7.9	15.5	13.2	34.4	<0.001	<0.001
	(3.8-9.7)	(4.8-12.3)	(8.2-32.8)	(6.7-47.5)	(16.4-50.7)		
Fibrinogen mg/dL	234.0	302.3	374.5	415.5	437.7	<0.001	<0.001
	(198-305)	(210.4-377.5)	(234-485.6)	(374-498)	(348.5-530.8)		

Note: Data represent median values and interquartile ranges. P value 1 was obtained using Kruskal-Wallis test, whereas P value 2 was calculated using Linear trend post test.

CRP: C-reactive protein.

**Table 4:** **Distribution of chemokines and other proteins levels in the study subjects stratified by *Plasmodium vivax *malaria clinical outcome**

**Biomaker**	**Endemic controls**	**Asymptomatic malaria**	**Symptomatic malaria**	**Severe malaria: Survivors**	**Severe malaria: Deaths**	**P value**	**P value**
						**1**	**2**
	**n=176**	**n=148**	**n=187**	**n=13**	**n=6**		
CCL2	86.0	85.2	65.8	64.7	87.9	0.117	0.207
ng/mL	(21-176)	(18-161.6)	(23.0-145.6)	(43-127)	(34.7-139)		
CCL5	27.0	24.4	25. 3	26.1	36.7	0.007	0.156
μg/mL	(15-45.7)	(13.4-38)	(15.8-70.2)	(20-38.7)	(25.4-83.2)		
CXCL9	0.3	0.5	2.2	3.5	4.6	<0.001	<0.001
ng/mL	(0.2-0.5)	(0.3-0.8)	(0.4-9.4)	(0. 6-12.5)	(0.8-9.9)		
CXCL10	88.0	19.4	77.4	117.7	183.3	<0.001	<0.001
pg/mL	(25-198)	(10.2-25.0)	(25.3-360.7)	(28-564)	(30.0-395.5)		
sTNF-RI	0.2	0.5	0.6	0.8	2.2	<0.001	<0.001
ng/mL	(0.1-0.4)	(0.4-0.6)	(0.4-0.7)	(0.7-0.9)	(1.7-3.1)		
SOD-1	4.2	6.0	26.0	72.6	82.4	<0.001	<0.001
ng/mL	(2.6-6.8)	(3.6-10)	(18.8-34.0)	(71-80.4)	(76.6-100.5)		
HO-1	29.1	29.5	35.5	42.9	49.2	<0.001	<0.001
ng/mL	(25.8-32)	(26.3-35.2)	(29.7-44.8)	(38.5-45)	(48.4-57.3)		

Note: Data represent median values and interquartile ranges. P value 1 was obtained using Kruskal-Wallis test, whereas P value 2 was calculated using Linear trend post test.

### Network analysis

The inferential network was generated from the values of each mediator measured in the plasma samples, in which it was observed that patterns of the concentrations and clinical classification of the subjects were based on the disease outcomes. The systemic levels of each mediator were input in the DimReduction software [18]. As a result, the DimReduction software selected the patterns of distribution of the mediators associated with each clinical group. Moreover, the same software was applied to identify links of interaction between the mediators. Following this approach, each mediator is selected as a target and the DimReduction software performs a search within the other mediators for those that are associated with the target in terms of entropy. As a result, the features related to the selected target are linked. This process is repeated by considering each mediator at a time and the result is the inferred network among the input values. The DimReduction default parameters values were kept fixed during all the experiments.

In order to analyse the structure of the biomarker networks, the network density was adopted, which is, in the context of this work, the ratio of the number of edges inferred in the network over total number of possible edges between all pairs of nodes. The density measure is defined as follows: density = L/(N (N-1)/2), in which L is the number of observed edges and N is the total number of the nodes in the network. The density is normalized, ranging between 0 (no edges in the network) and 1 (all possible edges presents).

### Statistical analysis

In the exploratory analysis of the data, frequency tables were constructed and the Chi-square test was applied to evaluate the association between qualitative variables. The quantitative variables were tested for Gaussian distribution within the total sample using D’Agostino and Pearson omnibus normality test. The variables with normal distribution were compared between the groups by one-way ANOVA with linear trend or Bonferroni’s multiple comparisons post-test. Further analysis were based on non-parametric tests only, considering the small number of individuals located in the two groups with severe vivax malaria. In this context, Kruskal Wallis test was used to assess the differences between the clinical groups and Mann-Kendall test was used to estimate liner trends. Only results from the non-parametric analyses are shown. Correlations between parasitaemia and the biomarkers from the networks were assessed using the Spearman rank test. For each analysis, P<0.05 was considered statistically significant. The graphics for the network analysis were customized using the Ingenuity Systems Pathway Analysis software (Ingenuity Systems, Redwood City, CA, USA). The statistical analyses were performed using the programs GraphPad Prism 5.0 (GraphPad Software Inc, USA), STATA 9.0 (StataCorp, TX, USA), and JMP 9.0 (SAS, Cary, NC, USA).

## Results

**Figure 1:**
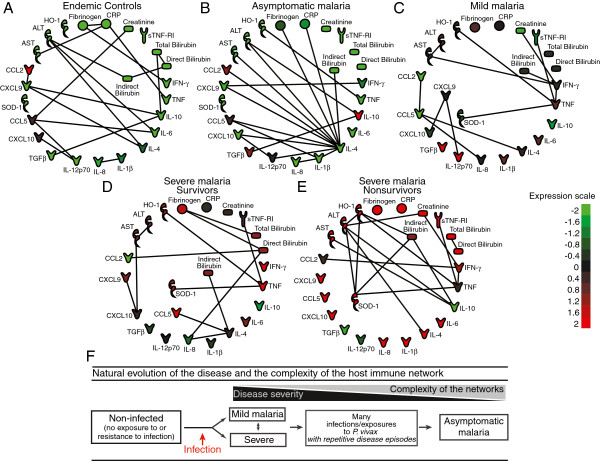
**Networks of candidate biomarkers during *Plasmodium vivax *malaria.** Plasma levels of several biomarkers of inflammation, tissue damage and oxidative stress were measured in 176 non-infected healthy individuals (**A**) and in 148 with asymptomatic infection (**B**), 187 with mild disease (**C**), 13 with severe non-lethal malaria (**D**) and six patients who died with *Plasmodium vivax* infection within seven days of hospitalization (**E**). The colours shown for each symbol represent the fold variation from the median values (log transformed) calculated for each marker. Each connecting line represents a significant interaction (P<0.05) detected by the network analysis using the DimReduction software. In (**F**), a summary model of the results with regard to the complexity of the networks in the context of the natural history of malaria is illustrated.

**Figure 2:**
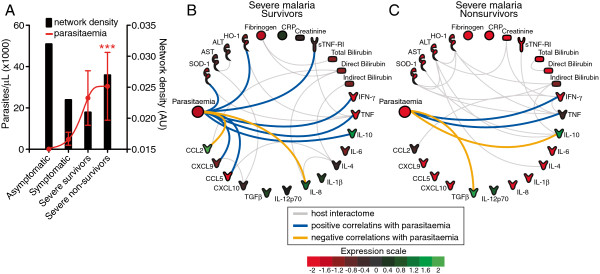
**Associations between *Plasmodium vivax *parasitaemia and host biomarkers.** (**A**) The distribution of *P. vivax* parasitaemia in different clinical groups is shown in red symbols (geometric means and 95% confidence intervals) whereas the values for network densities are shown as black bars. The variation of parasitaemia according to the malaria clinical severity was assessed using the Mann-Kendall test for linear trend (*** P<0.001). Statistically significant correlations between parasitaemia and different biomarkers are shown in (**B**) for the group of individuals with non-lethal severe malaria and in (**C**) for the group of patients that died during hospitalization. The correlations were assessed using the Spearman rank test. The correlations involving parasitaemia shown in (**B**) and (**C**) are plotted on top of the host interactome.

The plasma concentrations of most mediators measured were statistically different among the clinical groups, and many markers displayed linear trend to increase or decrease according to the degree of disease severity, as detailed in the Tables 2, 3 and 4. In the network analysis, densities of biomarker networks were observed, ranging between 0.021 and 0.032 in the different groups (Figure 1 and Figure 2A). The groups of non-infected individuals and those with asymptomatic malaria presented higher connectivity among the molecules, 0.030 and 0.032 respectively, compared to the groups of individuals with symptomatic infection (symptomatic malaria: 0.023; non-lethal severe disease: 0.021; and lethal malaria: 0.027) (Figure 2A).

In the group of non-infected individuals, CCL5 and CXCL9 had several significant interactions, and the latter was linked to the regulatory cytokines IL-10 and IL-4. In addition, CRP, ALT and IL-10 also presented relatively high connectivity, arguing that a fine tune of interactions involving these markers may influence the resistance against *P. vivax* infection. In those individuals in which infection did not lead to the onset of symptoms, the number of significant interactions was similar to the group of non-infected people (19 significant hits in the group of uninfected individuals and 18 hits in asymptomatic malaria), but the number of associations involving mainly IL-4 and to a lesser extent IL-1β was greatly increased in these individuals, leading to a totally different pattern of connectivity in the network (Figure 1).

In contrast, the patterns of connections between the mediators seen in the groups of individuals with symptomatic malaria were dramatically diverse than in the groups without the disease. The group of subjects with mild malaria displayed a substantial loss of the number of significant interactions in the network and the pro-inflammatory cytokines IFN-γ, TNF and CCL5 had the highest number of connections (Figure 1). IFN-γ was associated with indirect bilirubin, creatinine, AST and TNF, arguing that this cytokine could be driving inflammation (Figure 1). A number of other significant connections were detected between pro-inflammatory mediators, such as IL-12 and CXCL9. TNF-α was associated with IFN-γ, sTNF-RI, SOD-1 and HO-1, implying a potential interplay between those mediators in the pathogenesis of malaria-related symptoms. Intriguingly, the number of significant interactions and the overall complexity of networks were systematically reduced in the group of patients with severe forms of malaria (Figure 1). In the group of individuals who survived severe malaria, TNF, IL-4 and direct bilirubin seemed to have a distinguished pattern of connections, albeit, no clear clusters were evident and there was overall low connectivity between the markers studied. Moreover, in the group of individuals that succumbed to infection and died, TNF, ALT, HO-1, SOD-1, IL-10, IFN-γ and indirect bilirubin displayed more connectivity with other biomarkers, which caused a small increase in the complexity of the network when compared to the group of individuals with non-lethal severe malaria (Figure 1). Because *P. vivax* parasite counts in the blood displayed a strong linear trend to increase according to the disease clinical severity in the study population (Table 1 and Figure 2A), the potential associations between parasitaemia levels and the pattern of expression of the different biomarkers from the networks were investigated in the groups of patients with elevated morbidity. In the group of individuals presenting with severe non-lethal malaria, parasitaemia displayed significant positive associations with TNF, sTNF-RI, IFN-γ, IL-10, CXCL9, CXCL10, SOD-1 and HO-1, while negatively correlated with the chemokines IL-8 and CCL2 (Figure 2B). Interestingly, the number of significant associations involving parasitaemia was greatly reduced in the group of patients that died with severe vivax malaria (Figure 2C). The positive correlations involving TNF and IFN-γ persisted in this group of highly susceptible patients who died, while the regulatory cytokines IL-10 and TGF-β were negatively correlated with parasitaemia (Figure 2C).

## Discussion

The present study evaluated for the first time the density and complexity of the network of interactions between several markers strongly associated with host immune responses in their relationship with markers of tissue injury against *P. vivax* malaria in the context of the disease clinical outcomes. The results presented herein demonstrate that non-infected endemic controls exhibit a rich and dense network of interactions among immune-related markers and those involved in pathology. Such network is drastically deranged in symptomatic individuals but it is reconstituted in asymptomatic individuals. In this context it is stressed that asymptomatic infection is a state achieved by long-time residents in endemic areas and strongly related to several previous episodes of symptomatic malaria [6] being thus related to the development of a partial resistance to infection and/or tolerance to disease [19]. As a consequence, the onset of symptoms is prevented by limiting parasite burden and controlling inflammation. Such asymptomatic carriers develop just enough immunity to protect them from malarial illness, but not from malarial infection [1]. In the present study, asymptomatic carriers displayed the highest density values in the networks interactions, which argue that there may exist a complex regulatory mechanism behind the susceptibility to infection and tolerance and/or resistance against malaria. The network density values described in this study are similar to another protein-protein and metabolism-associated networks described previously in many other clinical contexts [20], which suggests that immune system may display similar homeostatic responses involving intrinsically complex regulatory pathways. Additional studies assessing network analysis of the immune responses in other infections and clinical contexts are needed to identify potential key common factors that could be targeted in a therapeutic approach.

The high connectivity exhibited by CXCL9 may be indicative of an important role for this chemokine in exposed but non-infected individuals since it correlates with protection against malaria in volunteers vaccinated by the circumsporozoite protein-based vaccine [21]. In addition, both IL-10 and IL-4 had significant connections with CXCL9, which also interacted with CCL2 and IFN-γ in those non-infected and with symptomless *P. vivax* infection, respectively. These interactions evidence a protective role of IL-4 and IL-10 through a modulatory effect on these pro-inflammatory mediators. Support for this modulatory hypothesis comes from the description that IL-10 and IL-4 can directly down regulate pro-inflammatory cytokines such as IL-6, TNF and IL-1β and prevent severe forms of malaria [22-26]. Studies with mouse macrophages *in vitro* argue that IL-4 suppresses the expression of CXCL9 and other IFN-γ inducible genes via STAT 6 signalling [27]. Although interesting, a role of a direct modulatory link between IL-10, IL-4 and CCL2 and/or CXCL9 and pro-inflammatory status of *Plasmodium* infected individuals is yet unexplored mechanistically and deserves further investigation.

Extensive research has been done in the identification of factors associated with severe forms of malaria, with significant contributions to the understanding of the disease pathogenesis. In the present study and not surprisingly, IFN-γ and TNF and the chemokine CCL5 were demonstrated to be crucial biomarkers in the network profile of those individuals with mild vivax malaria. CCL5 is a chemokine involved in the generation of inflammatory cellular infiltrates and its low levels are linked to cerebral malaria and severe malarial anaemia, maybe as consequence of thrombocytopaenia and the influence of haemozoin production [28-30]. The connectivity between IL-12 and CXCL9 seen in the networks from mildly symptomatic patients is also observed in the context of cancer therapy, in which administration of IL-12 increases the expression of CXCL9 in peripheral blood mononuclear cells as a response against the tumour [31,32]. Interactions involving CXCL9 had an intriguing pattern between the study groups. This chemokine was more associated with regulatory cytokines in the group of non-infected controls, which may suggest a potential influence over the resistance to infection. On the other side, CXCL9 was linked to pro-inflammatory IL-12 within symptomatic subjects, which may be associated with the onset of clinical disease.

While networking the immune response in lethal cases of severe *P. vivax* malaria, it was found an important role for HO-1, an enzyme primarily responsible for the host detoxification from harmful free haem and which produces carbon monoxide (CO), free iron and biliverdin. Higher HO-1 levels were described in patients with symptomatic vivax malaria and this enzyme may contribute (both very high and low levels) to disease severity [8,33]. Lower expression of HO-1 may result in greater availability of free haem resulting in severe malaria, while high expression of HO-1 could lead to increased synthesis of free iron, which also can be harmful as can promote oxidative damage [33]. Furthermore, SOD-1, previously associated with *P. vivax* malaria [7], displayed connectivity only in the groups of patients with symptomatic disease, especially in those individuals who died upon infection. Accumulation of SOD-1 without a compensatory elevation of catalase of glutathione enzymes could lead to accumulation of H_2_O_2_ and the production of more free radicals by Fenton’s reaction [34]. Indeed, it has been described that both glutathione and catalase might be reduced during severe malaria [35] and high levels of SOD-1 could lead to the exacerbation of the oxidative stress and inflammation, aggravating the outcome of the disease. In addition, the release of haem during malaria erythrocytic cycle reduces the production of anti-inflammatory prostaglandin E_2_ and TGF-β from mononuclear cells via SOD-1 [16]. Thus, SOD-1 connectivity seen herein in severe malaria patients seems to be a response against the high production of deleterious haem during the robust intravascular haemolysis observed in severe malaria.

Furthermore, TNF, IFN-γ and IL-10 also had important interactions in individuals who succumbed to malaria infection. IL-10 seems to modulate Th1-type responses to *Plasmodium* antigens by downregulating TNF, and IFN-γ levels by dampening the release of IL-12p70, thus resulting in an impaired immune response that may lead to lethality [5,36]. However, it has also been described that in cases of uncontrolled inflammation with high production of pro-inflammatory mediators may be associated with exhaustion of the protective regulatory responses [6]. In response to malaria infection, the patterns of expression of multiple cytokines and the relative balance and interactions between these factors may be capable of mediating protective immunity or disease severity depending of the context [37]. These findings infer that fine-tuning between all the mediators is extremely important to prevent malaria lethality.

A major observation from the analysis of the groups of patients with mild or severe malaria was that the overall complexity of the networks by means of a number of significant interactions was reduced while compared to what was seen in healthy subjects. Intriguingly, in the group of individuals with asymptomatic malaria, the complexity of the network was restored and several significant interactions were detected, but with a clear central role for IL-4. The potential anti-inflammatory effect of IL-4 in the immune response during malaria is not yet fully understood [38]. IL-4 seems to assist in antisporozoite immunity and studies in mice infected with *Plasmodium yoelii* suggest that IL-4 is also required for the development of CD8+ T lymphocytes [39] and for the development of a memory response against liver stage parasite [40]. Furthermore, IL-4 interferes with the maturation of Th1 cells and can reduce the production of IFN-γ in some experimental settings [41]. The high number of interactions between IL-4 and others biomarkers in asymptomatic individuals highlights a possible importance of this molecule in modulating the disease tolerance of individuals constantly exposed to *P. vivax* and further investigations should address in detail the importance of IL-4 in symptomless malaria. A summary of the results with regard to the complexity of the network connectivity in the context of the natural evolution of vivax malaria is shown in Figure 1F.

In the groups of patients with severe malaria, *P. vivax* parasitaemia displayed several positive interactions with previously described inflammatory mediators that participate in the immunopathogenesis of malaria, such as TNF, IFN-γ and IL-10 [1,6,7] in addition to TGF-β. Surprisingly, liver transaminases, bilirubins, creatinine and CRP were not correlated with parasitaemia. The lack of direct interactions between parasitaemia and laboratory surrogates of inflammation-driven tissue injury and oxidative stress suggests that the parasite burden may influence more stringently the host immune response rather than induce lethal tissue damage itself. This idea, if validated in other studies, reinforces the argument that susceptibility of disease severity in vivax malaria might be a case of dysfunction of the host homeostatic system caused by an inflammatory imbalance driven by lack of resistance against *P. vivax*.

## Conclusion

In summary, the systematic analysis of several mediators of inflammation measured simultaneously was able to characterize the overall pattern of immune response of patients with *P. vivax* malaria according to disease severity and clinical outcome. This approach also revealed other levels of complexity of the disease, involving intricate associations between unique markers, such as TNF, IFN-γ, IL-4, HO-1 and SOD-1. IL-4, despite being at low systemic levels, seems to be a central mediator with several significant interactions in individuals with asymptomatic malaria. In addition, connectivity involving CXCL9 and IFN-γ was more prevalent in the groups of individuals non-infected or with mild manifestations of the disease while associations involving SOD-1 and HO-1 were more evident in more severe and lethal cases. In severe malaria, parasitaemia seems to positively associate with pro-inflammatory mediators, and the simultaneous negative correlations involving the regulatory cytokines IL-10 and TGF-β argue that there is a deregulated balance of the host immune response that is skewed towards amplification of inflammation. Networking analysis may represent a potential tool to understand the interactions between several mediators in the context of malarial disease severity. The identification of critical factors driving malaria pathogenesis can guide future therapeutic approaches.

## Abbreviations

(SOD)-1: Superoxide dismutase;(HO)-1: Haem oxygenase;FUNASA: Brazilian national foundation of health;PCR: Polymerase chain reaction;AST: Aspartate amino-transferase;ALT: Alanine amino-transaminase;CRP: C-reactive protein;CBA: Cytometric bead array;sTNF-RI: TNF receptor I;(TGF)-β: Transforming growth factor;CO: Carbon monoxide

## Competing interests

The authors declare that they have no competing interests.

## Authors’ contributions

VRRM helped interpreting the data and to write the manuscript together with BBA and MB-N. AQ and FML performed the network analysis. BBA conceptualized the study, coordinated the clinical assessments and performed the laboratory experiments, analysed the data and wrote the first draft of the manuscript. MB-N supervised the clinical study and helped with data interpretation and the writing of the manuscript. All authors have read and approved the final version of the manuscript.
